# Prostate Secretory Protein of 94 Amino Acids (PSP94) Binds to Prostatic Acid Phosphatase (PAP) in Human Seminal Plasma

**DOI:** 10.1371/journal.pone.0058631

**Published:** 2013-03-04

**Authors:** Jenifer H. Anklesaria, Dhanashree D. Jagtap, Bhakti R. Pathak, Kaushiki M. Kadam, Shaini Joseph, Smita D. Mahale

**Affiliations:** 1 Division of Structural Biology, National Institute for Research in Reproductive Health, Parel, Mumbai, India; 2 Proteomics Facility, National Institute for Research in Reproductive Health, Parel, Mumbai, India; 3 ICMR Biomedical Informatics Centre, National Institute for Research in Reproductive Health, Parel, Mumbai, India; Gentofte University Hospital, Denmark

## Abstract

Prostate Secretory Protein of 94 amino acids (PSP94) is one of the major proteins present in the human seminal plasma. Though several functions have been predicted for this protein, its exact role either in sperm function or in prostate pathophysiology has not been clearly defined. Attempts to understand the mechanism of action of PSP94 has led to the search for its probable binding partners. This has resulted in the identification of PSP94 binding proteins in plasma and seminal plasma from human. During the chromatographic separation step of proteins from human seminal plasma by reversed phase HPLC, we had observed that in addition to the main fraction of PSP94, other fractions containing higher molecular weight proteins also showed the presence of detectable amounts of PSP94. This prompted us to hypothesize that PSP94 could be present in the seminal plasma complexed with other protein/s of higher molecular weight. One such fraction containing a major protein of ∼47 kDa, on characterization by mass spectrometric analysis, was identified to be Prostatic Acid Phosphatase (PAP). The ability of PAP present in this fraction to bind to PSP94 was demonstrated by affinity chromatography. Co-immunoprecipitation experiments confirmed the presence of PSP94-PAP complex both in the fraction studied and in the fresh seminal plasma. In silico molecular modeling of the PSP94-PAP complex suggests that β-strands 1 and 6 of PSP94 appear to interact with domain 2 of PAP, while β-strands 7 and 10 with domain 1 of PAP. This is the first report which suggests that PSP94 can bind to PAP and the PAP-bound PSP94 is present in human seminal plasma.

## Introduction

Prostate Secretory Protein of 94 amino acids (PSP94) is secreted by the epithelial cells of the prostate [1] and is one of the major constituents present in human seminal plasma [2]. It is a non-glycosylated, cysteine rich protein with a theoretical molecular mass of 10.7 kDa, which on SDS-PAGE (Sodium Dodecyl Sulfate-Polyacrylamide Gel Electrophoresis) shows an apparent molecular mass of ∼17 kDa [3]. PSP94 was earlier referred to as human seminal plasma inhibin (HSPI) [4] or beta-microseminoprotein (β-MSP) [5]. Presence of PSP94 in other reproductive as well as some non-reproductive tissues, both in males and females has been previously reported [6]–[8]. Though this protein has been well characterized structurally [9], [10], its exact function and mechanism of action have not been unequivocally established. Postulated biological functions of PSP94 in males with respect to sperm function include inhibition of sperm motility [11] and prevention of spontaneous acrosome reaction in sperm [12]. During prostate tumorigenesis, PSP94 levels have been observed to decrease [13]. It is not clear whether these reduced levels of PSP94 play any role during prostate cancer progression. Efforts have been made by several researchers to demonstrate the clinical potential of serum levels of PSP94 as a diagnostic marker for prostate cancer [14], [15]. However, the observation that PSP94 is present in free as well as bound form in the serum has made it difficult to accurately correlate PSP94 levels with prostate cancer [16], [17]. Recent studies have also shown PSP94 to possess calcium and pH dependent candidacidal activity [18].

Previous studies by our group and others have shown that PSP94 has the ability to bind to human immunoglobulin [19], [20]. It has been hypothesized that high amounts of PSP94 present in the seminal plasma would bind to immunoglobulin and prevent an immune response to spermatozoa in the female reproductive tract [21]. In order to understand the biological significance of PSP94, an approach of identifying and characterizing its putative binding proteins has been employed. Towards this, PSP94 binding protein (PSPBP) from human plasma has been reported to interact with PSP94 [22]. The N-terminal SCP (sperm coating protein) domain of PSPBP has been proposed to be the PSP94 interaction site. Based on these findings, another protein, CRISP-3 (Cysteine Rich Secretory Protein-3) present in the seminal plasma, also comprising of the SCP domain was identified as a PSP94 interacting protein [23].

During the isolation and purification of PSP94 from human seminal plasma [19], we observed that the fractions collected subsequent to PSP94 containing fraction continued to show detectable levels of PSP94 during the reversed phase - high performance liquid chromatography (RP-HPLC) purification step. We hypothesized that PSP94 may be present in the bound form with other proteins in these fractions. One such protein capable of interacting with PSP94 was identified and characterized to be Prostatic Acid Phosphatase (PAP) and the details are presented in this paper.

## Materials and Methods

### Fractionation of Human Seminal Plasma Proteins

The present study was approved by the NIRRH Ethics Committee for Clinical Studies. Waiver of informed consent was granted as anonymised, left over semen samples after completion of the laboratory tests (only those tested as normal) were used in the present study. The collection and analysis of the ejaculates were performed according to World Health Organization recommendations [24]. The samples were obtained in a sterile plastic tube following physical examination of the ejaculate (pH, volume, sperm concentration, total motility and sperm morphology). Semen samples showing a sperm concentration of ≥15×10^6^, total motility of >40% and sperm morphology of >4% normal forms were included in the study. The samples (approximately 2 ml/individual) were centrifuged at 3000 g for 20 min and the seminal plasma was stored at –80°C. The method employed for the purification of PSP94 from human seminal plasma has been described earlier [19]. Briefly, seminal plasma was subjected to ammonium sulphate precipitation and Phenyl Sepharose chromatography. This was followed by preparative RP-HPLC on a C-18 column (BioRad Laboratories, Hercules, CA, USA) using a linear gradient of 0% to 70% acetonitrile in 0.1% TFA (Trifluoroacetic acid)/H_2_O over a period of 3 h. The fractions obtained were analyzed by Matrix-Assisted Laser Desorption/Ionisation-Time Of Flight Mass Spectrometry (MALDI-TOF MS) (ABI SCIEX 4700 Mass Analyzer, Foster City, CA, USA) to determine the molecular mass of the constituents present in each of them. PSP94 was found to be the major constituent of fraction I and characterization of the same has been reported earlier [19]. Fractions II and III were further subjected to SDS-PAGE.

### One-Dimensional Gel Electrophoresis and Western Blot

Fractions I (10 ng), II (10 µg) and III (10 µg) were resolved on 12.5% SDS-PAGE under reducing conditions, followed by transfer on to a nitrocellulose membrane. After blocking the membrane in 5% non-fat dry milk (NFDM), it was incubated with affinity-purified rabbit polyclonal anti-human PSP94 antibody raised in the laboratory [19], at 1∶2000 dilution in TBS-T buffer (20 mM TBS, pH 7.4 containing 0.1% Tween 20 in 1% NFDM), for 1 h at room temperature (RT). The blot was further incubated with horseradish-peroxidase conjugated polyclonal goat anti-rabbit secondary antibody (Bangalore Genei, Bangalore, India), at 1∶2000 dilution in TBS-T buffer, for 1 h at RT. Finally, the blot was developed using enhanced chemiluminescence (ECL Plus, GE Healthcare, Buckinghamshire, UK).

Fractions I (1 µg), II (10 µg) and III (10 µg) resolved on 12.5% SDS-PAGE gel were stained with silver nitrate. Upon silver staining, the major band (∼47 kDa) of fraction III was excised from the gel and subjected to trypsin digestion for protein identification using MALDI-TOF/TOF mass spectrometry as described earlier [25]. The peptides were analyzed in MS as well as MS/MS mode to obtain sequence specific information. The resulting peptide mass fingerprints were searched against the NCBI (National Center for Biotechnology Information)/SwissProt database using MASCOT search engine.

Fraction III (20 µg) was then resolved on a 12.5% SDS-PAGE and subjected to immunoblot analysis using mouse monoclonal anti-PAP antibody (US Biological, Swampscott, MA), at 1∶2000 dilution in TBS-T buffer, for 1 h at RT. The blot was then incubated in horseradish-peroxidase conjugated polyclonal goat anti-mouse secondary antibody (Santa Cruz Biotechnology, CA, USA), at 1∶2000 dilution in TBS-T buffer, for 1 h at RT. The blot was then developed with ECL Plus reagent.

### Two-Dimensional Gel Electrophoresis

Fraction III (100 µg) of preparative RP-HPLC was further subjected to isoeletric focusing (IEF) for the first-dimension according to the protocol described by Tanu et al. [26]. The proteins were resolved in the second dimension on 12.5% SDS-PAGE and stained with silver nitrate. The protein spots were manually excised and further processed for MALDI-TOF/TOF analysis (as described previously).

### Capturing of PSP94 Interacting Proteins from Fraction II and Fraction III

PSP94 was conjugated to CNBr-activated Sepharose 4B beads (GE Healthcare Bio-Sciences AB) as described below. PSP94 (2 mg) purified and characterized in the laboratory [19] was dissolved in 1 ml of coupling buffer (100 mM Na_2_CO_3_, pH 8.3, 0.5 M NaCl) and incubated with 1 ml of swollen CNBr-activated Sepharose beads (in a final gel to buffer ratio of 1∶2 v/v) overnight at 4°C on an end-to-end shaker. After coupling, the unreacted groups were blocked with 1 M ethanolamine-HCl, pH 8.5 for 2 h at RT. Finally, the PSP94 conjugated Sepharose beads were washed and suspended in PBS buffer (10 mM, pH 7.2). Next, fraction II and III (20 µg/300 µl of 50 mM PBS, pH 7.2 buffer containing 0.5% Triton X-100) were incubated separately with 75 µl of non-conjugated beads for 2 h at 37°C on an end-to-end shaker, followed by centrifugation. After pre-clearing, the supernatant was incubated with 75 µl of PSP94 conjugated Sepharose beads for 2 h at 37°C on an end-to-end shaker. The reaction was carried out in screw cap columns (Thermo Fisher Scientific, Rockford, USA) and at the end of the incubation period, the column was washed with PBS-TX100 to remove unbound proteins and the last wash was collected. The bound protein (eluate) was eluted with 0.2 M glycine-HCl (pH 2.8) and neutralized with 1 M Tris (pH 9.5). The same volume of the eluate and last wash (to be used as negative control) were concentrated to a fixed volume by ultrafiltration using a 3 kDa molecular weight cut-off membrane (Millipore, Cork, Ireland).

### Identification and Characterization of PSP94 Bound Protein

The concentrated eluate and the last wash obtained from the column of PSP94 conjugated Sepharose beads were subjected to 12.5% SDS-PAGE under reducing conditions (in two sets). One of the eluate lanes was stained with silver nitrate and the major band observed was subjected to tryptic digestion, followed by MALDI-TOF/TOF mass spectrometry for protein identification. The input (10 µg of fraction II and III respectively) as positive control (lane 1) along with the last wash as a negative control (lane 2) and the eluate (lane 3) were subjected to SDS-PAGE followed by immunoblot analysis and probed with the antibody of the respective protein identified in silver staining. In case of fraction II, anti-hCRISP-3 goat polyclonal antibody (R&D Systems Inc., Minneapolis, MN, USA), at 1∶1000 dilution was used, followed by incubation with horseradish peroxidase-conjugated rabbit anti-goat secondary antibody (Santa Cruz Biotech Inc.), at 1∶2000 dilution. In case of fraction III, mouse monoclonal anti-PAP antibody at 1∶2000 dilution was used, followed by horseradish-peroxidase conjugated polyclonal goat anti-mouse secondary antibody, at 1∶2000 dilution.

### Co-Immunoprecipitation of PSP94 and PAP

To demonstrate the presence of PSP94-PAP complex in fraction III, co-immunoprecipitation experiments were carried out. Fraction III (50 µg) in 500 µl PBS-TX100 buffer was incubated with 20 µl of Protein G Sepharose beads (GE Healthcare Bio-Sciences AB) for 30 min at 4°C with end-to-end shaking, followed by centrifugation. After pre-clearing, the supernatant was immunoprecipitated with anti-PAP antibody or mouse IgG2b isotype control antibody (Sigma, Missouri, USA) by incubating with 20 µl of fresh Protein G Sepharose beads overnight at 4°C with end-to-end mixing. The beads were washed thoroughly with PBS-TX100 buffer and then boiled in reducing sample buffer for 5 min. The proteins were resolved on 12.5% SDS-PAGE and transferred on to a nitrocellulose membrane for immunoblot analysis with anti-PSP94 antibody (as described previously). Parallel experiments were performed, wherein anti-PSP94 antibody or normal rabbit serum was used for immunoprecipitation and anti-PAP antibody was used for immunoblot analysis. Another experiment was carried out to show the interaction of pure PSP94 (500 ng) with pure PAP (US Biological, Swampscott, MA) (500 ng) in vitro. In this, anti-PAP antibody was used for immunoprecipitation and anti-PSP94 antibody (1∶4000 dilution) was used for immunoblotting. A separate set of experiments were carried out to detect the presence of PSP94-PAP protein complex in human seminal plasma (100 µg) using seminal plasma diluted in PBS and the same procedure was followed.

### Molecular Modeling of PSP94-PAP Complex

Three dimensional structures of human PSP94 (PDB ID: 3IX0; chain B) and human PAP (PDB ID: 1ND6; chain A) were used for generating the model of PSP94-PAP complex. Molecular modeling was done using Discovery Studio 2.0 (Accelrys Inc.) software. The water molecules in the crystal structure were removed and hydrogen atoms were added to both the structures. The Protein Utilities tool was used to correct any problems in the structures. The structures were energy minimized using 200 steps of steepest descent algorithm. PSP94 and PAP structures were docked using the ZDOCK algorithm and the poses generated were grouped into 50 clusters with RMSD and interface cutoff of 10 Å. Further refinement of the docked structures was done using the Refine Docked Proteins (RDOCK) protocol. The best pose was selected based on the ZDOCK score and RDOCK energy values.

## Results

### Fractionation of Human Seminal Plasma Proteins

**Figure 1:**
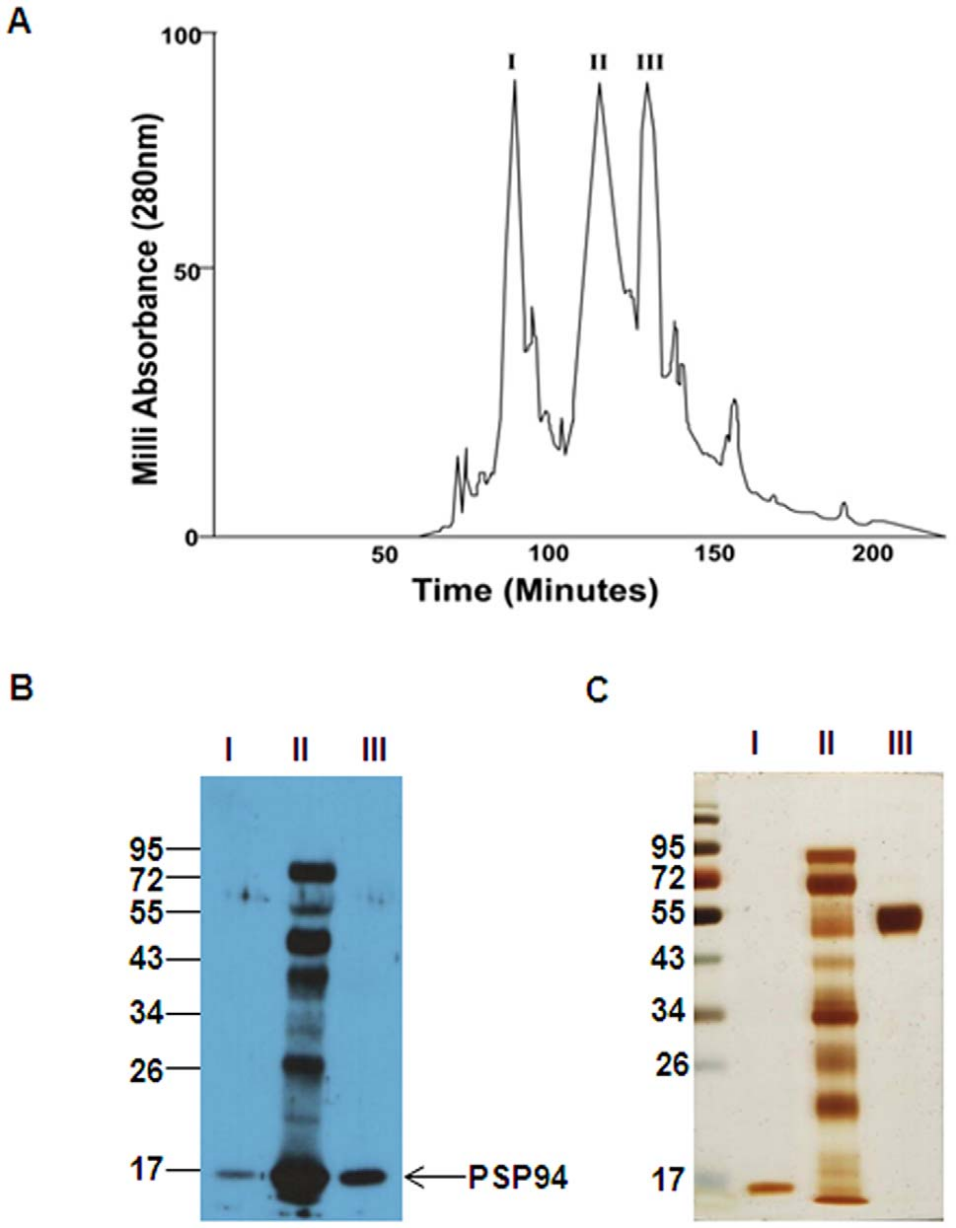
Fractionation of human seminal plasma proteins. **A.** Preparative RP-HPLC profile of seminal plasma proteins (bound fraction from Phenyl Sepharose chromatography) showing three major peaks (fractions I, II and III). **B.** Immunoblot of fractions I, II and III probed with anti-PSP94 antibody. **C.** One dimensional SDS-PAGE profile of fractions I, II and III. Molecular weight markers shown are in kDa.

Human seminal plasma was fractionated in three steps using ammonium sulphate precipitation, Phenyl Sepharose chromatography and preparative RP-HPLC. Three major peaks (fractions I, II and III) were obtained by preparative RP-HPLC (Figure 1A).

### One-Dimensional Gel Electrophoresis and Western Blot

**Figure 2:**
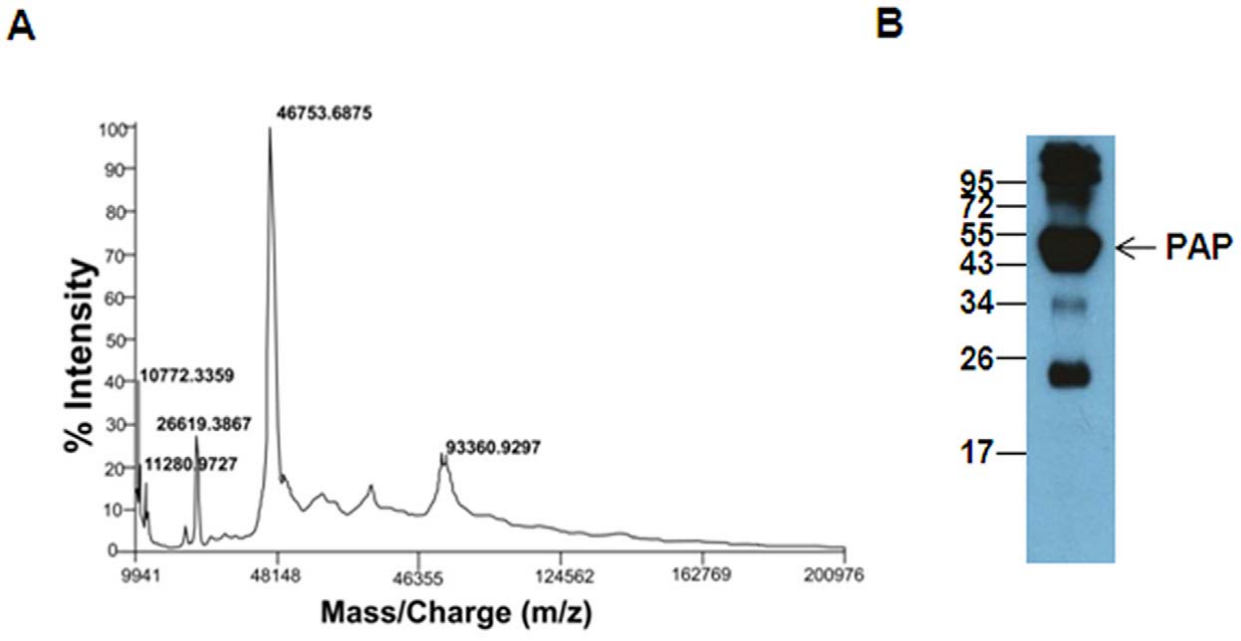
Identification of proteins in fraction III. **A.** MALDI-TOF mass spectra of fraction III from preparative RP-HPLC showing a major peak of molecular mass 46753 Da. The peak at 10772 Da is probably of PSP94. **B.** Immunoblot analysis of fraction III probed with anti-PAP antibody. Molecular weight markers shown are in kDa.

To characterize the proteins in the fractions, these were further subjected to SDS-PAGE followed by immunoblotting using anti-PSP94 antibody (Figure 1B). Fraction I has been characterized earlier and was found to be PSP94 [19]. Immunoblot of fractions II and III using anti-PSP94 antibody, showed the presence of an immunoreactive band at ∼17 kDa corresponding to PSP94. The complete protein profiles of fraction I, II and III were visualized on staining with silver nitrate (Figure 1C). MS analysis of fraction II showed a major peak of molecular mass 11.732 kDa and two minor peaks at 26.018 kDa and 28.118 kDa (data not shown). MS analysis of fraction III showed a major peak of molecular mass 46.753 kDa and a minor peak of mass 10.772 kDa which corresponds to the molecular weight of PSP94 (Figure 2A). The band corresponding to molecular weight of ∼47 kDa observed on the silver nitrate stained gel of fraction III (Figure 1C) was subjected to trypsin digestion followed by MS and MS/MS analysis. It was identified to be prostatic acid phosphatase precursor (Accession Number: gi|6382064) of human origin, having a mowse score of 276 with 39% peptide coverage (see Figure S1). The molecular mass and pI of the matched protein was found to be 44.880 kDa and 5.83 respectively. Fraction III when resolved on SDS-PAGE followed by immunoblotting using anti-PAP antibody showed immunoreactive bands of PAP at molecular weight of ∼47 kDa and ∼24 kDa (Figure 2B). In addition to these, immunoreactive bands at ∼34 kDa and above 95 kDa were also observed.

### Two-Dimensional PAGE of Fraction III

**Figure 3:**
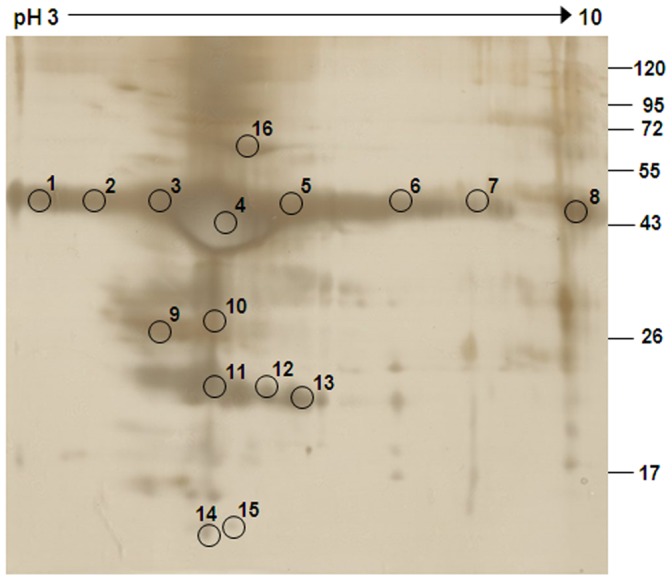
Two-dimensional gel electrophoresis profile of fraction III. The 2D gel resolved by isoelectricfocusing in the first dimension (pH range 3–10) followed by SDS-PAGE in the second dimension was stained with silver nitrate. The protein spots (circled) were excised and subjected to MS and MS/MS analysis (Data shown in Table 1). Molecular weight markers shown are in kDa.

For better resolution of proteins present in fraction III, two-dimensional gel electrophoresis was performed. On isoelectrofocusing of fraction III using pH 3–10 IPG strips, proteins between the molecular weight range of 20 to 55 kDa and in the pI range of 4 to 6 were observed (Figure 3). The protein spots were cored; trypsin digested and identified using MS and MS/MS analysis. Table 1 represents the proteins identified by MS/MS analysis with the accession number, isoelectric point (pI), molecular weight (MW), mowse score and percentage sequence coverage of the matched protein. The proteins corresponding to the molecular weight of ∼47 kDa (spots 1 to 8) and 24 kDa (spots 11, 12 and 13) were identified to be isoforms of PAP. Spots 9 and 10 (∼28 kDa) were identified to be carboxypeptidase E; while spots 14 and 15 (∼14 kDa) were found to be transthyretin amyloidogenic variants and spot 16 (∼68 kDa) was serum albumin.

**Table 1:** Mass spectrometric analysis of spots excised from the 2D gel.

Spot No.	Protein Identity and Accession Number	pI*	MW* (Da)	Mowse Score	% SequenceCoverage
1	Acid phosphatase, prostate (gi|14250150)	5.93	44829	408	47
2	Acid phosphatase, prostate (gi|16740983)	5.89	44854	146	42
3	Prostatic acid phosphatase precursor (gi|6382064)	5.83	44880	488	40
4	Acid phosphatase, prostate (gi|14250150)	5.93	44829	405	48
5	Acid phosphatase (gi|189619)	5.93	44910	437	46
6	Acid phosphatase, prostate (gi|16740983)	5.89	44854	149	44
7	Acid phosphatase (gi|189619)	5.93	44910	105	31
8	Prostatic acid phosphatase precursor (gi|6382064)	5.83	44880	419	43
9	Carboxypeptidase E (gi|6429043)	5.07	53601	466	39
10	Carboxypeptidase E (gi|6429043)	5.07	53601	71	41
11	Acid phosphatase (gi|189619)	5.93	44910	85	39
12	Acid phosphatase, prostate (gi|14250150)	5.93	44829	122	40
13	Acid phosphatase, prostate (gi|16740983)	5.89	44854	130	33
14	Chain A, Tertiary Structures Of Three AmyloidogenicTransthyretin Variants (gi|2098255)	5.57	13806	96	100
15	Chain A, Tertiary Structures Of Three AmyloidogenicTransthyretin Variants (gi|2098255)	5.57	13806	61	86
16	Chain A, Crystal Structure Of Human SerumAlbumin (gi|3212456)	5.67	68425	537	80

* Note: Discrepancy in the isoelectric point (pI) or molecular weight (MW) of the proteins detected here with that of the protein entries in NCBI database could be probably due to the post-translational modifications or protein processing.

### Identification and Characterization of Protein from PSP94 Affinity Chromatography

**Figure 4:**
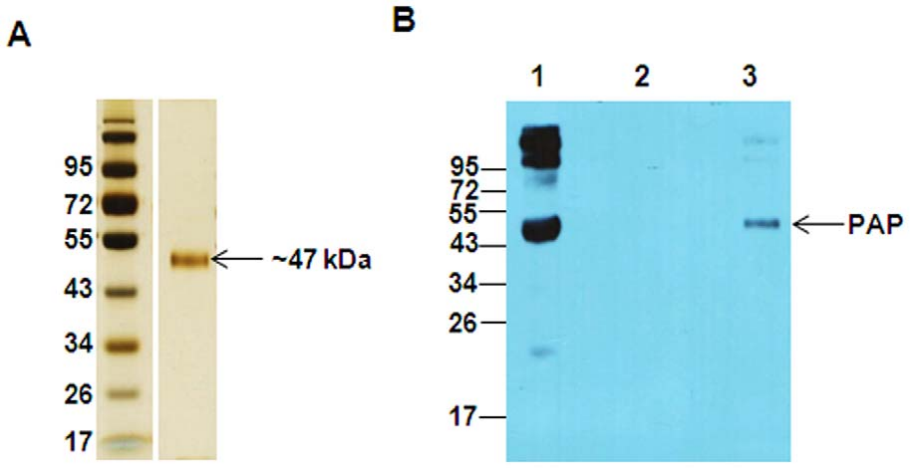
Identification and characterization of affinity purified PSP94 binding protein from fraction III. **A.** Gel stained with silver nitrate showing the presence of a band at ∼47 kDa in the eluate lane, further identified to be PAP protein on MS and MS/MS analysis (Figure S2). **B.** Immunoblot probed with anti-PAP antibody showing a band at ∼47 kDa in eluate (lane 3) corresponding to the band of immunoreactive PAP protein detected in the input (lane 1; 10 µg). The last wash (lane 2) did not show any band. Molecular weight markers shown are in kDa.

To identify the potential binding protein of PSP94, fraction III was passed through a PSP94 affinity column. The eluate obtained was concentrated and resolved on SDS-PAGE and stained with silver nitrate (Figure 4A). On staining, the eluate showed an intense band at molecular weight of ∼47 kDa, which was excised manually and subjected to MS and MS/MS analysis of its trypsin digested peptides for protein identification. This was identified as prostatic acid phosphatase precursor (Accession Number: gi|6382064) of human origin, having a mowse score of 125 with 39% peptide coverage (see Figure S2). The molecular mass and pI of the matched protein was found to be 44.880 kDa and 5.83 respectively. The 47 kDa band of the eluate was clearly detected as immunoreactive PAP on immunblotting with anti-PAP antibody (Figure 4B). Similarly, Fraction II when passed through the PSP94 affinity column, CRISP-3 was identified as one of the proteins in the eluate (see Figure S3). Since CRISP-3 is a well-documented binding partner of PSP94, our interest was to identify and characterize the PSP94 binding protein from Fraction III.

### Detection of PSP94-PAP Complex in Fraction III

**Figure 5:**
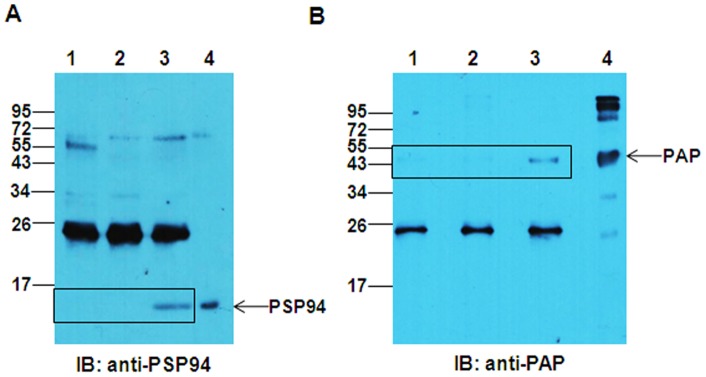
Co-immunoprecipitation of PSP94 and PAP proteins from fraction III. **A.** PSP94-PAP complex from fraction III (50 µg) co-immunoprecipitated with anti-PAP antibody showing the presence of PSP94 (lane 3). Protein G beads incubated with either fraction III in buffer alone (lane 2) or fraction III incubated with mouse isotype control antibody (lane 1) served as controls. 10 µg of fraction III was loaded in lane 4 as input and the immunoblot was probed with anti-PSP94 antibody. **B.** PSP94-PAP complex from fraction III (50 µg) was co-immunoprecipitated with anti-PSP94 antibody showing the presence of PAP (lane 3). Protein G beads incubated with either fraction III in buffer alone (lane 2) or fraction III incubated with normal rabbit serum (lane 1) served as controls. 10 µg of fraction III was loaded in lane 3 as input and the immunoblot was probed with anti-PAP antibody. Molecular weight markers shown are in kDa.

Co-immunoprecipitation experiments were performed to detect the presence of PSP94-PAP complex in fraction III. This complex was immunoprecipitated either with anti-PAP or anti-PSP94 antibody. The immunoprecipitated proteins were resolved on SDS-PAGE and analysed by immunoblotting with either anti-PSP94 or anti-PAP antibody (Figure 5A and Figure 5B respectively). It was observed that PSP94 immunoprecipitates with anti-PAP antibody (lane 3; Figure 5A), but not with mouse isotype control antibody (lane 1; Figure 5A) While PAP immunoprecipitated with anti-PSP94 antibody (lane 3; Figure 5B), but not with normal rabbit serum (lane 1; Figure 5B), indicating that PSP94-PAP complex is present in fraction III. For the negative control (lane 2; Figure 5A and Figure 5B), buffer was used in place of antibody during the immunoprecipitation step to rule out the possibility of non-specific binding of PSP94-PAP complex to Protein G beads. 10 µg of fraction III was loaded as input in lane 3 of both the blots. The intense bands observed in lane 3, predominantly at ∼26 kDa along with the higher molecular weight bands, could be non-specific as they were observed in the control lanes as well.

### Interaction of PSP94-PAP in vitro

**Figure 6:**
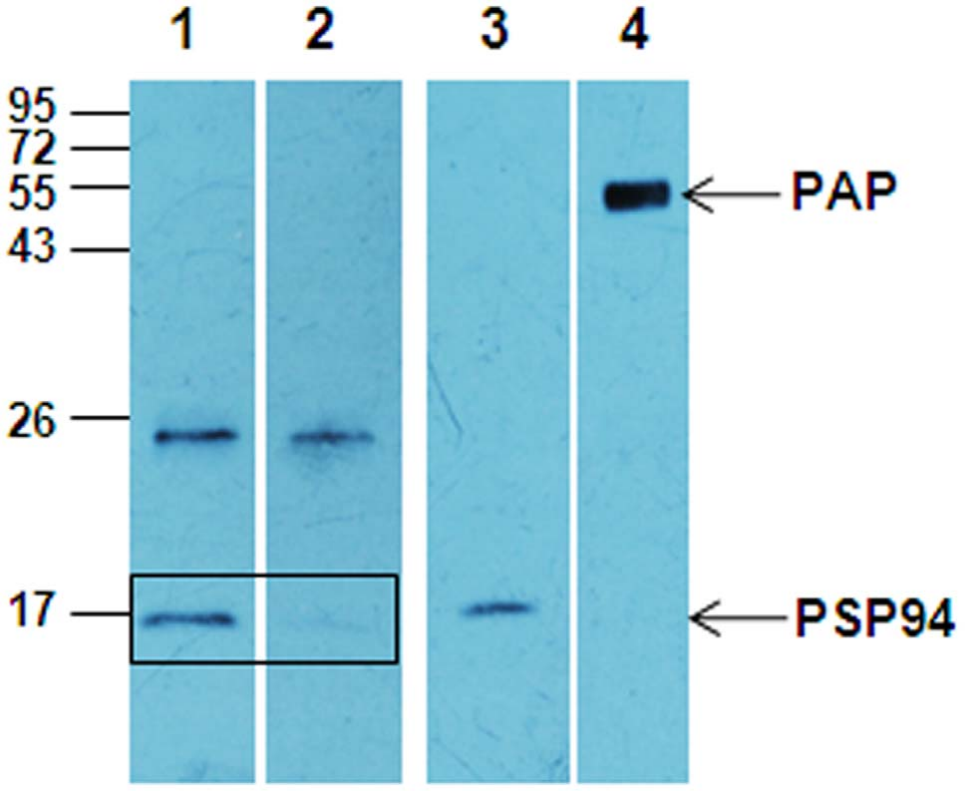
Interaction of pure PSP94 and PAP proteins in vitro. 500 ng of PSP94 incubated with or without PAP (500 ng) (lane 1 and 2 respectively) and immunoprecipitated using anti-PAP antibody. Pure PSP94 (20 ng; lane 3) and PAP (500 ng; lane 4) proteins were loaded as input. Lanes 1, 2 and 3 were immunoblotted with anti-PSP94 antibody, while lane 4 was immunoblotted with anti-PAP antibody. The immunoreactive band of PSP94 (∼17 kDa) is detected only in lane 1 and not in lane 2. Molecular weight markers shown are in kDa.

In order to confirm the interaction of PSP94 and PAP, pure proteins were pre-incubated and then subjected to immunoprecipitation with anti-PAP antibody, followed by immunoblotting with anti-PSP94 antibody (Figure 6). PSP94 immunoreactive band was observed in lane 1, demonstrating that PSP94 co-immunoprecipitates with PAP. In a separate tube, when PSP94 in the absence of PAP was used in the immunoprecipitation step with anti-PAP antibody, the band corresponding to PSP94 was absent (lane 2; Figure 6). Lanes 3 and 4 were the input for PSP94 and PAP proteins respectively. Other non-specific bands were also seen in lane 1 as observed in the earlier experiment.

### Detection of PSP94-PAP Complex in Human Seminal Plasma

**Figure 7:**
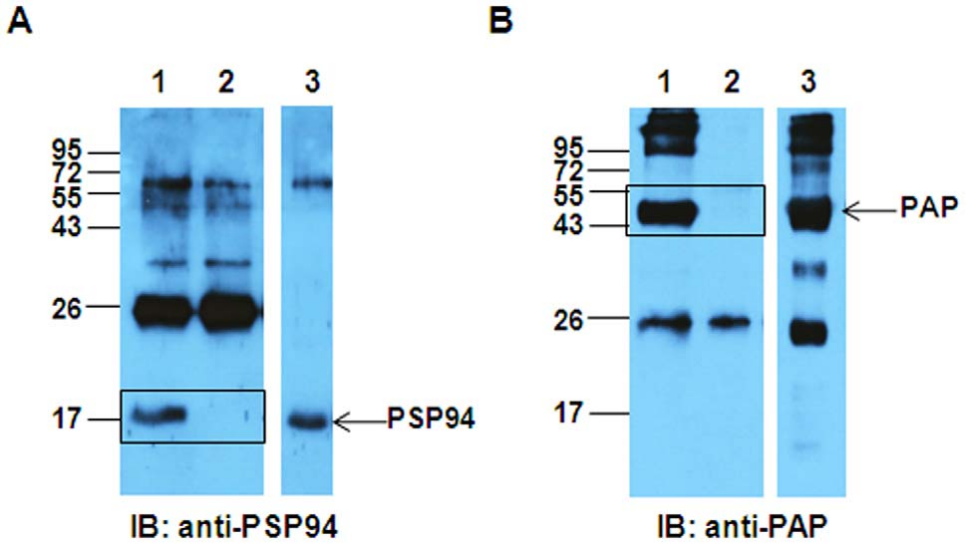
Detection of PSP94-PAP complex in human seminal plasma. **A.** PSP94-PAP complex from seminal plasma (100 µg) was co-immunoprecipitated with anti-PAP antibody (lane 1). Protein G beads incubated with seminal plasma in buffer alone served as the negative control (lane 2). 20 µg of seminal plasma was loaded in lane 3 as input and the immunoblot was probed with anti-PSP94 antibody. The immunoreactive band of PSP94 (∼17 kDa) is detected only in lane 1 and not in lane 2. **B.** PSP94-PAP complex from seminal plasma (100 µg) was co-immunoprecipitated with anti-PSP94 antibody (lane 1). Protein G beads incubated with seminal plasma in buffer alone served as the negative control (lane 2). 20 µg of seminal plasma was loaded in lane 3 as input and the immunoblot was probed with anti-PAP antibody. The immunoreactive band of PAP (∼47 kDa) is detected only in lane 1 and not in lane 2. Molecular weight markers shown are in kDa.

In order to establish that the interaction between PSP94 and PAP proteins occurs naturally under physiological conditions, co-immunoprecipitation studies were carried out with normal human seminal plasma sample (Figure 7). Immunoprecipitation of proteins from seminal plasma using anti-PAP antibody and immunoblotting with anti-PSP94 antibody showed the presence of ∼17 kDa immunoreactive PSP94 band (lane 1; Figure 7A). Similarly, immunoprecipitation of seminal plasma using anti-PSP94 antibody and immunoblotting with anti-PAP antibody, showed the presence of ∼47 kDa immunoreactive PAP band (lane 1; Figure 7B), indicating that PSP94 is also present in bound to PAP is present in seminal plasma. Respective buffer controls did not show the presence of PSP94 or PAP (lane 2; Figure 7A and Figure 7B respectively). 20 µg of seminal plasma was loaded as input in lane 3 of both the blots. The origin of the other bands recognised in addition to the specific band in lane 1 is unknown and may be non-specific as they were observed in the control lane as well.

### Molecular Model of PSP94-PAP Complex

To understand the nature of interaction between PSP94 and PAP proteins, in silico docking studies were performed. The refined docked complex of PSP94 and PAP (Figure S4) gave a ZDOCK score of 17.12 with a RDOCK energy of −19.177 kcal/mol. In the proposed model of PSP94-PAP complex, beta sheets 1 and 6 of PSP94 appear to interact with domain 2 of PAP, while beta sheets 7 and 10 of PSP94 appear to interact with domain 1 of PAP. The interactions between PSP94 and PAP obtained from the Protein Interactions Calculator (PIC) webserver [27] are listed in Table S1 (see Table S1).

## Discussion

PSP94 secreted by the prostatic epithelial cells is one of the most abundant proteins present in human seminal plasma. Numerous biological roles have been hypothesized for this protein; however its exact function still remains elusive. Recent efforts have been focused towards identifying the binding partners of PSP94 in order to gain knowledge of its biological interactions. This has led to the identification of PSP94 binding proteins in serum and in seminal plasma. PSP94 has been demonstrated to interact with a protein called PSPBP from human blood plasma [22] and CRISP-3 present in human seminal plasma [23]. Besides PSPBP and CRISP-3, PSP94 has also been shown to bind to human immunoglobulin [19], [20]. Our recent studies on structural elucidation of PSP94 revealed that two PSP94 monomers can associate to form a homodimer [10]. Based on the findings so far, it can be inferred that PSP94 may exist either as a homodimer or a heterodimer complexed with its interacting proteins. The concentration of PSP94 (600–900 mg/L) [2] is many fold higher than that of CRISP-3 (3–30 mg/L) in seminal plasma [28], this indicates that a significant amount of PSP94 could exist either in the free form or bound to any other protein in a complex.

The present study for the first time identifies prostatic acid phosphatase (PAP) as a binding partner of PSP94 in human seminal plasma. During the process of isolation and purification of PSP94 from human seminal plasma, we observed that, in addition to the main fraction containing PSP94 (fraction I), two other fractions (fraction II and fraction III) also showed the presence of detectable levels of immunoreactive PSP94 (Figure 1). This indicated that fractions II and III could contain PSP94 in a bound form. Instead of using neat whole seminal plasma which contains excess PSP94, we used partially purified fractions separated from PSP94 in the present study. Of these, fraction II revealed the presence of multiple PSP94 immunoreactive bands (in lane II of Figure 1B). However, the affinity pull down assay identified only CRISP-3 as the major binding partner (Figure S3) in fraction II. On further characterization of fraction III, MS and MS/MS analysis revealed PAP to be one of its major constituents (Figure 2). Presence of PAP together with PSP94 in fraction III indicated that PSP94 and PAP may exist in a bound form in the seminal plasma.

Fraction III was further subjected to two-dimensional gel electrophoresis to resolve its component proteins. The 2D gel profile revealed multiple spots (1 to 8) at ∼47 kDa, however, in the pI range of 4 to 6 (Figure 3). These spots on MS/MS analysis were identified to be PAP (Table 1). Earlier reports by Starita-Geribaldi et al. [29] have shown that PAP appears as a cluster with average molecular weight of around 45 kDa with the same pI range as observed by us. Other reports also suggest that secretory PAP exists as multiple isoforms, which mainly differ in their glycosylation pattern [30], [31]. MS/MS analysis of other protein spots also identified lower molecular weight forms of PAP (spots 11–13); along with carboxypeptidase E (spots 9 and 10) and transthyretin amyloidogenic variants (spots 14 and 15). Presence of lower molecular weight fragments of PAP, together with carboxypeptidase E in the seminal plasma has also been reported by Marquínez et al. [32]. Similarly, naturally occurring fragments of PAP are found to be present as amyloid fibrils in the seminal plasma [33].

The potential binding protein of PSP94 from fraction III was pulled down using PSP94 conjugated beads which on MS/MS analysis was identified to be PAP (Figure 4). We demonstrated PSP94-PAP protein interaction in fraction III by performing co-immunoprecipitation experiments (Figure 5). Higher molecular weight immunoreactive bands (at ∼68 kDa) detected in this experiment was speculated to be that of serum albumin (as identified by 2D gel electrophoresis). The corresponding band from silver stained gel when subjected to mass spectrometric analysis did not show the presence of albumin (data not shown). Besides, if albumin were to act as a co-binding protein for PSP94 and PAP, we would have expected it to be detected in the eluate from the affinity pull down as well, but only a single band at ∼47 kDa was observed (Figure 4). To further validate PSP94-PAP interaction, we performed similar co-immunoprecipitation experiments using pure PSP94 and PAP proteins (Figure 6). The physiological existence of PSP94-PAP protein complexes was investigated using fresh human seminal plasma; wherein PSP94 was co-immunoprecipitated with PAP (Figure 7). This indicated that PSP94 is naturally complexed with its binding protein within human seminal plasma. It also confirmed the possible existence of PSP94 present in PAP-bound form in the seminal plasma. The intense band at ∼26 kDa observed in all the co-immunoprecipitation experiments was initially believed to be the light chain of the antibody used for immunoprecipitation. However, it is observed in the buffer control lane as well, thus indicating it to be a non-specific band of unknown origin.

In silico docking studies were undertaken to delineate the interaction between PSP94 and PAP. Our crystal structure studies reveal that PSP94 can exist as a homodimer [10]. We have also shown that at acidic pH, PSP94 can dissociate into monomers [10] suggesting that free PSP94 can be available for interaction with other proteins. Based on the studies of PSP94-CRISP-3 interaction [34] and the models generated for PSP94-CRISP-3 and PSP94-immunoglobulin complex [10], [21], PSP94 appears to interact through its terminal β-strands. Our present data on molecular modeling studies indicate that PAP can also interact with PSP94 through the terminal beta sheets of PSP94.

PAP is one of the predominant proteins secreted by the epithelial cells of the prostate [2] and is present in high amounts (1000–2000 mg/L) in human semen [35]. It is a highly glycosylated enzyme and is reported to be present either in the intracellular form or as the secretory form [36], [37]. In the seminal plasma, lysophosphatidic acid has been shown to be degraded due to the lipid phosphatase activity of PAP [38]. Also, other studies have demonstrated that, PAP along with PSA (Prostate-Specific Antigen) participates in the proteolysis of semenogelins (seminal coagulum forming proteins) leading to the liquefaction of human semen [39]. Previous reports indicate that only the dimeric form of hPAP has full catalytic activity and the dissociation of dimer to monomers results in protein inactivation [40]. From our studies, PAP appears to be a potential binding protein of PSP94. Whether PSP94 can prevent the formation of PAP dimer is not known yet and it would be interesting to study if PSP94 has any role in PAP function.

Based on the data presented here and previously published work, PSP94 has been shown to interact with PAP and CRISP-3 proteins present in seminal plasma. Recent studies on other seminal plasma protein complexes, like the eppin protein complex (EPC), have suggested the existence of a network of protein-protein interactions, consisting of central proteins that have more than one interaction within a complex which may be conserved and essential for function [41]. Similarly, a PAP-containing zinc-binding multiprotein complex has also been characterized from the human seminal plasma [42]. Whether PSP94 is a part of such a functional network needs to be investigated further. In summary, our results indicate that PSP94 interacts with PAP and PSP94-PAP complexes are present in the seminal plasma.

## Supporting Information

Figure S1 **Amino acid sequence of the 47 kDa band from lane III of** **Figure 1C** **.** The matched protein corresponds to prostatic acid phosphatase precursor, wherein the underlined region represents the peptides identified on MS/MS analysis searched against the NCBI database. The amino acid sequence from 1 to 32 corresponds to the signal peptide.
(TIF)

Figure S2 **Amino acid sequence of the 47 kDa band** **from the eluate lane of** **Figure 4A** **.** The matched protein corresponds to prostatic acid phosphatase precursor, wherein the underlined region represents the peptides identified on MS/MS analysis searched against the NCBI database. The amino acid sequence from 1 to 32 corresponds to the signal peptide.
(TIF)

Figure S3 **Identification and characterization of affinity purified PSP94 binding protein from fraction II.** **A.** Gel stained with silver nitrate showing the presence of a band at ∼26 kDa and ∼28 kDa in the eluate lane. **B.** Immunoblot probed with anti-hCRISP3 antibody showing a band at ∼26 kDa and ∼28 kDa in eluate (lane 3) corresponding to the band of immunoreactive CRISP-3 protein detected in the input (lane 1; 10 µg). The last wash (lane 2) did not show any band. Molecular weight markers shown are in kDa. **C.** Amino acid sequence of the 26 kDa band (from the eluate lane of the silver nitrate stained gel; Figure S3A) which corresponds to Cysteine-rich secretory protein 3, wherein the underlined region represents the peptides identified on MS/MS analysis searched against the SwissProt database. The amino acid sequence from 1 to 20 corresponds to the signal peptide.
(TIF)

Figure S4 **A proposed model of PSP94-PAP complex.** PSP94 (cyan) docked with PAP (brown) showing the binding interfaces of PSP94 and PAP highlighted in pink and green respectively. Right panel shows another view rotated by 180° around the vertical axis.
(TIF)

Table S1 **Residues from PSP94 and PAP involved in the interaction as per the modeled structure shown in Figure S4.**
(DOC)
