# Supplementary figures and images for: Prostate Secretory Protein of 94 Amino Acids (PSP94) Binds to Prostatic Acid Phosphatase (PAP) in Human Seminal Plasma
Source: PLoS One. 2013 Mar 4;8(3):e58631. doi: 10.1371/journal.pone.0058631 (PMC3587604; doi:10.1371/journal.pone.0058631)

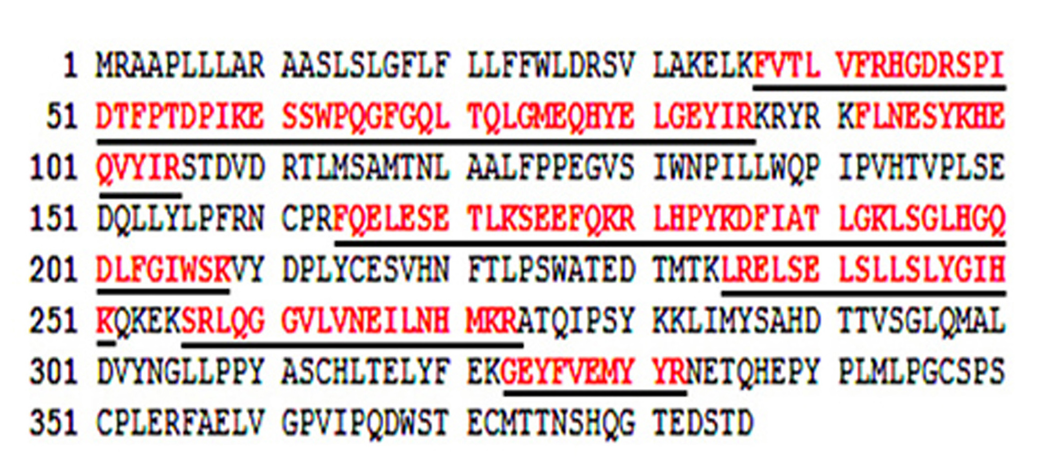

Supplement: Figure S1 — Amino acid sequence of the 47 kDa band from lane III of Figure 1C . The matched protein corresponds to prostatic acid phosphatase precursor, wherein the underlined region represents the peptides identified on MS/MS analysis searched against the NCBI database. The amino acid sequence from 1 to 32 corresponds to the signal peptide. (TIF) [file pone.0058631.s001.tif]

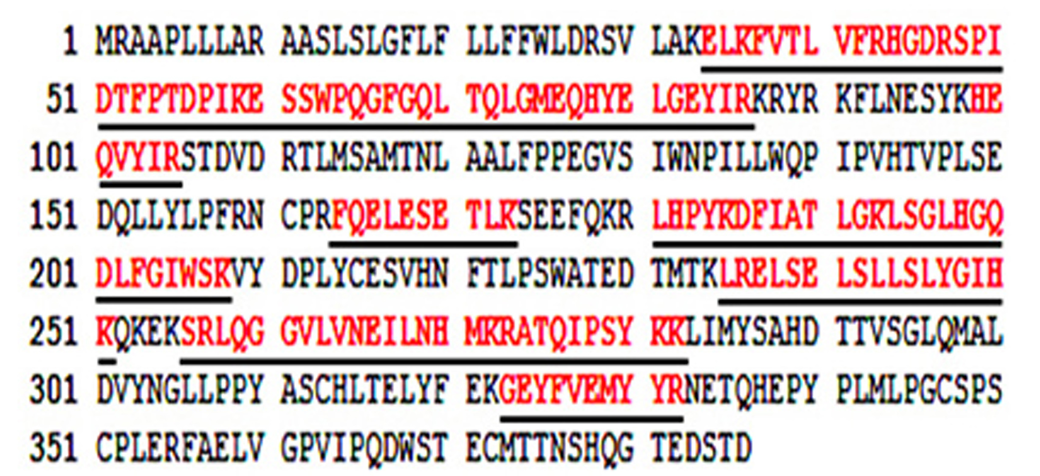

Supplement: Figure S2 — Amino acid sequence of the 47 kDa band from the eluate lane of Figure 4A . The matched protein corresponds to prostatic acid phosphatase precursor, wherein the underlined region represents the peptides identified on MS/MS analysis searched against the NCBI database. The amino acid sequence from 1 to 32 corresponds to the signal peptide. (TIF) [file pone.0058631.s002.tif]

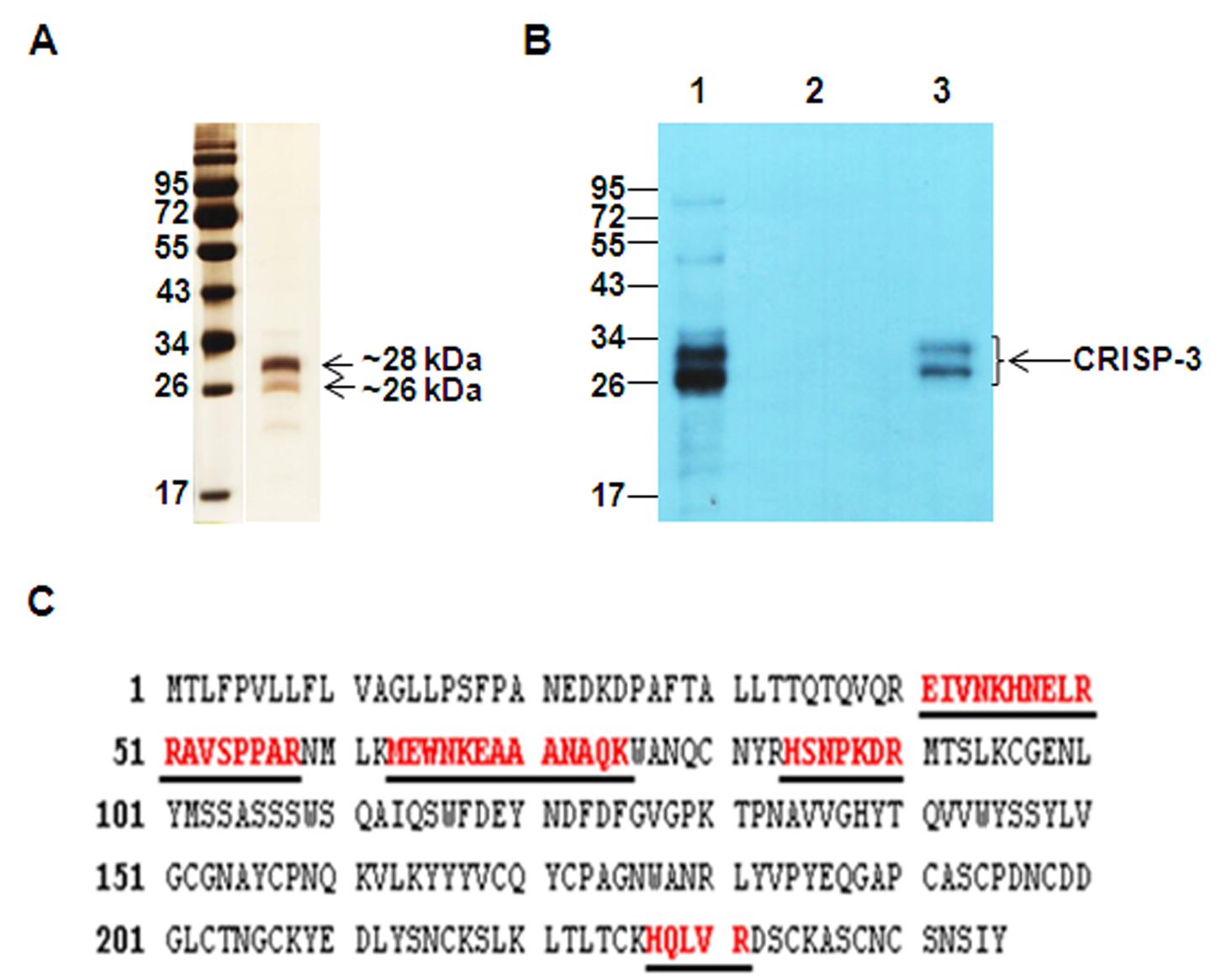

Supplement: Figure S3 — Identification and characterization of affinity purified PSP94 binding protein from fraction II. A. Gel stained with silver nitrate showing the presence of a band at ∼26 kDa and ∼28 kDa in the eluate lane. B. Immunoblot probed with anti-hCRISP3 antibody showing a band at ∼26 kDa and ∼28 kDa in eluate (lane 3) corresponding to the band of immunoreactive CRISP-3 protein detected in the input (lane 1; 10 µg). The last wash (lane 2) did not show any band. Molecular weight markers shown are in kDa. C. Amino acid sequence of the 26 kDa band (from the eluate lane of the silver nitrate stained gel; Figure S3A) which corresponds to Cysteine-rich secretory protein 3, wherein the underlined region represents the peptides identified on MS/MS analysis searched against the SwissProt database. The amino acid sequence from 1 to 20 corresponds to the signal peptide. (TIF) [file pone.0058631.s003.tif]

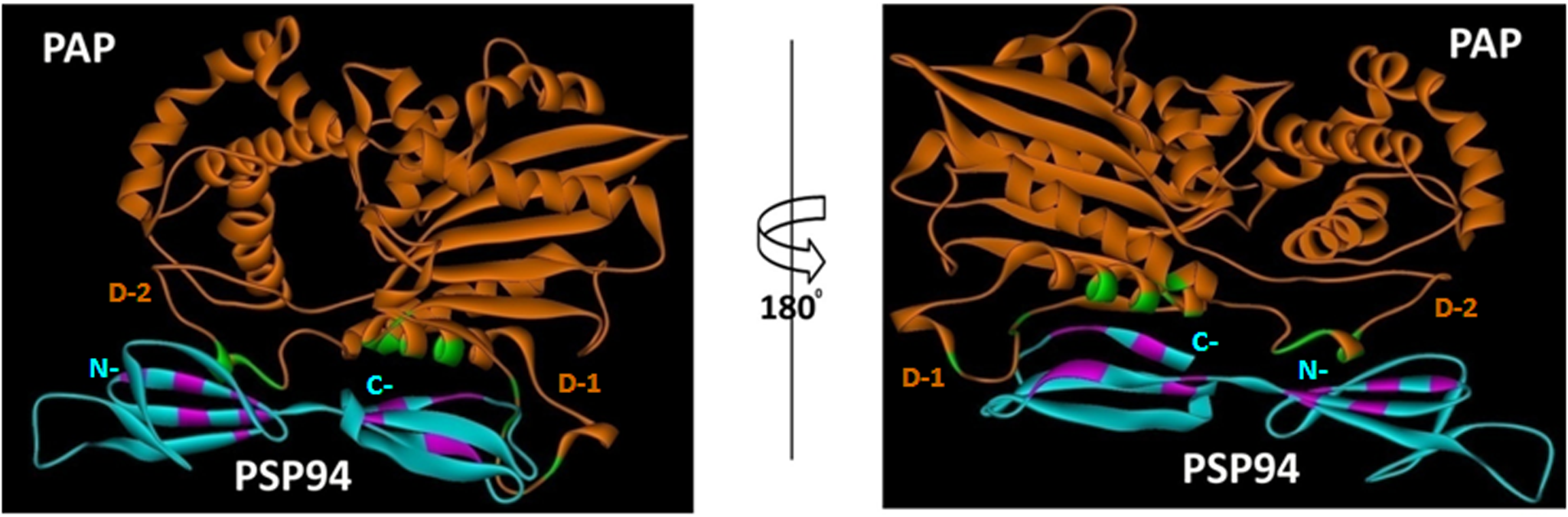

Supplement: Figure S4 — A proposed model of PSP94-PAP complex. PSP94 (cyan) docked with PAP (brown) showing the binding interfaces of PSP94 and PAP highlighted in pink and green respectively. Right panel shows another view rotated by 180° around the vertical axis. (TIF) [file pone.0058631.s004.tif]
