# Supplementary material for: Prostate Secretory Protein of 94 Amino Acids (PSP94) Binds to Prostatic Acid Phosphatase (PAP) in Human Seminal Plasma
Source: PLoS One. 2013 Mar 4;8(3):e58631. doi: 10.1371/journal.pone.0058631 (PMC3587604; doi:10.1371/journal.pone.0058631)
Supplement: Table S1 — Residues from PSP94 and PAP involved in the interaction as per the modeled structure shown in Figure S4. (DOC) [file pone.0058631.s005.doc]

Table S1. Residues from PSP94 and PAP involved in the interaction as per the modeled structure shown in Figure S4.

| **Type of interaction** | **PSP94 residue** | **PAP residue** |
| --- | --- | --- |
| **Hydrophobic interactions within 5 Å** | Tyr59  Val89 | Trp106  Trp106 |
|  | Val89 | Met85 |
|  | Trp92 | Met43 |
|  | Trp92 | Met82 |
|  | Trp92 | Met85 |
| **Hydrogen bonds** | Cys2 | Ser29 |
|  | Cys2 | Trp31 |
|  | Cys2 | Pro32 |
|  | Cys50 | Glu28 |
|  | Cys50 | Ser29 |
|  | Cys50 | Trp31 |
|  | Cys50 | Pro32 |
|  | Ser88 | Gln107 |
|  | Ser88 | Ile109 |
|  | Val89 | Met85 |
|  | Trp92 | Met43 |
|  | Ser90 | Thr86 |
|  | Gly58 | Trp106 |
|  | Ser88 | Trp106 |
|  | Ser88 | Ile109 |
|  | Thr39 | Glu28 |
|  | Thr39 | Glu28 |
|  | Thr41 | Ser29 |
|  | Ser48 | Ser29 |
|  | Ser54 | Met82 |
|  | Lys74 | Asp76 |
|  | Lys74 | Asp76 |
|  | Tyr43 | Ser29 |
|  | Ser48 | Ser29 |
|  | Asp60 | Trp100 |
| **Ionic interactions within 6 Å** | Lys74 | Asp76 |
|  | Lys74 | Asp78 |
| **Aromatic-aromatic interactions within 4.5-7 Å** | Tyr59 | Trp106 |
| **Aromatic-sulphur interactions within 5.3 Å** | Trp92 | Met82 |
